# The Kidney Clock Contributes to Timekeeping by the Master Circadian Clock

**DOI:** 10.3390/ijms20112765

**Published:** 2019-06-05

**Authors:** Jihwan Myung, Mei-Yi Wu, Chun-Ya Lee, Amalia Ridla Rahim, Vuong Hung Truong, Dean Wu, Hugh David Piggins, Mai-Szu Wu

**Affiliations:** 1 Graduate Institute of Mind, Brain and Consciousness, Taipei Medical University, Taipei 11031, Taiwan; amaliaridla@gmail.com (A.R.R.); truonghungvuong@hotmail.com (V.H.T.); 2 Brain and Consciousness Research Center, Taipei Medical University-Shuang Ho Hospital, New Taipei City 23561, Taiwan; 3 Laboratory of Braintime, Taipei Medical University, Taipei 11031 & Shuang Ho Hospital, New Taipei City 23561, Taiwan; s9150921@hotmail.com; 4 Computational Neuroscience Unit, Okinawa Institute of Science and Technology, Okinawa 904-0495, Japan; 5 Division of Nephrology, Department of Internal Medicine, Shuang Ho Hospital, Taipei Medical University, Taipei 11031, Taiwan; e220121@gmail.com; 6 Division of Nephrology, Department of Internal Medicine, School of Medicine, College of Medicine, Taipei Medical University, Taipei 11031, Taiwan; 7 Institute of Epidemiology and Preventive Medicine, College of Public Health, National Taiwan University, Taipei 10672, Taiwan; 8 Department of Neurology, Shuang Ho Hospital, New Taipei City 23561, Taiwan; tingyu02139@gmail.com; 9 Department of Neurology, Taipei Medical University, Taipei 11031, Taiwan; 10 School of Physiology, Pharmacology, and Neuroscience, Faculty of Life Sciences, University of Bristol, Biomedical Sciences Building, University Walk, Bristol BS8 1TD, UK; hugh.piggins@bristol.ac.uk

**Keywords:** circadian clocks, systemic clocks, CKD, kidney, SCN, hierarchical organization

## Abstract

The kidney harbors one of the strongest circadian clocks in the body. Kidney failure has long been known to cause circadian sleep disturbances. Using an adenine-induced model of chronic kidney disease (CKD) in mice, we probe the possibility that such sleep disturbances originate from aberrant circadian rhythms in kidney. Under the CKD condition, mice developed unstable behavioral circadian rhythms. When observed in isolation in vitro, the pacing of the master clock, the suprachiasmatic nucleus (SCN), remained uncompromised, while the kidney clock became a less robust circadian oscillator with a longer period. We find this analogous to the silencing of a strong slave clock in the brain, the choroid plexus, which alters the pacing of the SCN. We propose that the kidney also contributes to overall circadian timekeeping at the whole-body level, through bottom-up feedback in the hierarchical structure of the mammalian circadian clocks.

## 1. Introduction

Circadian rhythms of the body have been thought to be controlled by a central pacemaker; in mammals, the suprachiasmatic nucleus (SCN). Through numerous ablation studies, the SCN became recognized as the brain’s functional clock module [1]. Endogenous generation of clock signals from the SCN has been demonstrated by implantation [2] and by single-cell-level measurement studies [3]. However, it was found that the SCN is not the only endogenous clock [4] and transgenic gene expression reporter systems have revealed further organizations of circadian clocks in the peripheral systems [5,6]. The brain too contains several other endogenous clocks [7] and it was recently found that the choroid plexus (CP) clock exceeds even the SCN clock in terms of robustness [8]. These investigations have recast the role of the SCN as the master *coordinator* of the rest of the circadian clocks of the body, rather than the master pacemaker. Daily changes of ambient light register upon the SCN directly from retina [9] and the SCN actively gates the environmental cycle with its own clock before entraining other organs [10]. Such a hierarchical view of circadian clocks has been successful as a first description of the systems-level organization [11,12]. However, this view was challenged by the discovery of a liver circadian clock, which can be independently entrained by feeding rhythm [13]. Although a hierarchical organization suggests a one-way flow of information, biological systems often have a bi-directional signaling structure. In the circadian oscillator system of the brain, the CP clock participates in determining the circadian period of behavioral locomotor activities by speeding up the SCN clock [8]. This contribution of peripheral clocks to central timekeeping is probably replicated in other circadian clocks in the body.

Outside the brain, the kidney maintains the second most robust circadian gene expression rhythms, next to the liver [14]. Circadian rhythms of renal functional parameters have been well-established [15]. Many genes that determine renal functions are expressed in a circadian manner, the rhythm of which becomes impaired by circadian clock disruption [16]. Bioluminescence reporter imaging for expression of the core circadian gene *Per2* (PER2::LUC) in cultured kidney slices shows a strong circadian rhythm of clock gene expression, which also regulates circadian osmolarity rhythm in explants [17]. Although the kidney receives efferent input from the brain [18], which can include circadian signals from the SCN, dialysis can disturb circadian rhythmicity of sleep [19]. Aberrant sleep phases are common, especially among end-stage renal disease (ESRD) patients. Their unstable sleep patterns may reflect alterations in intrinsic sleep mechanism, but they may also reflect destabilized circadian rhythms. In nephrectomy rats, alterations in sleep architecture as well as circadian clock gene expression have been noted under normal light–dark conditions [20]. 

To clarify effects of kidney failure on circadian rhythms, we used an adenine-induced renal failure model in mice. Continuous feeding of a low-dose adenine diet can induce renal failure while leaving other organs relatively intact, making it a non-surgical model of chronic kidney disease (CKD) [21,22]. The adenine diet model produces rapid-onset kidney disease with extensive tubulointerstitial fibrosis, and tubular atrophy.

We found that under mild dosage (0.2% *w*/*w* adenine diet), aberrant circadian rhythmicity can occur in mice. These nocturnal animals gradually developed disorganized locomotor bouts during subjective night time and/or a destabilized circadian period. They also showed severe signs of chronic inflammation in the kidney, both histologically and in terms of blood biochemistry. If the local circadian clock in kidney (“kidney clock”) influences the SCN’s circadian timekeeping in vivo, we can postulate that a damaged clock in kidney under CKD would incorrectly feed back to the SCN and cause the unstable behavioral rhythms. Under isolation in vitro, CKD kidneys showed unstable and longer period circadian rhythms while the SCN rhythms remain intact. Since the SCN regulates the behavioral circadian rhythms, and as it remains intact under CKD, the observed behavioral changes in vivo must come from some internal feedback influences from outside the SCN. The strongest clocks outside the brain are liver and kidney, and we found that the adenine-induced damage is severe in kidney, and not liver. It is difficult to establish the mechanism by which the kidney clock signals the SCN but these observations do provide initial correlational evidence that such kidney-to-SCN feedback does exist. We postulate that the rhythmic readjustment of the fluid homeostasis by the kidney affects the SCN clock, and alters circadian rhythms at the behavioral level.

## 2. Results

### 2.1. Weak and Disorganized Circadian Rhythmicity in CKD Mice

**Figure 1:**
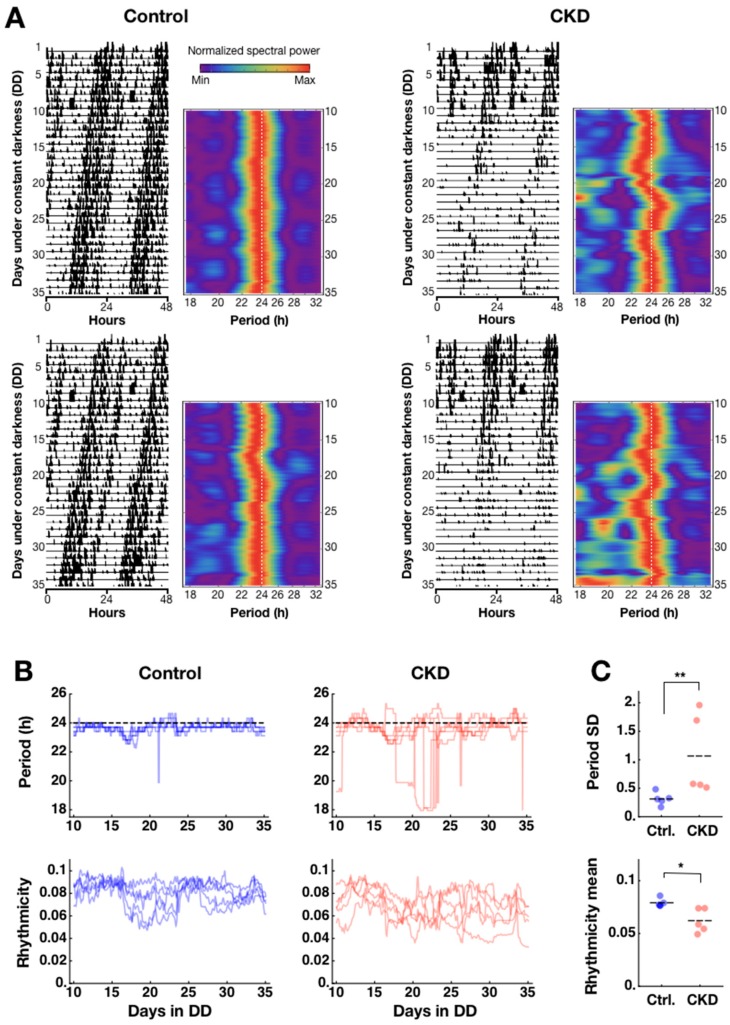
Adenine-induced chronic kidney disease (CKD) impairs circadian locomotor activity rhythm. (**A**) Circadian locomotor activities are shown side-by-side in doubleplots and spectrograms for two representative control mice (left panel) and CKD mice (right panel). Doubleplots were aligned to the actual local time of recording, as indicated on the *x*-axis (0, 24, and 48 correspond to 0:00 h of each day). Adenine was given to the CKD group from day 1. The spectrogram was normalized at each time point. The first 10 days were not included due to initial jitters. (**B**) (Upper) In control mice, the period remained stable (*n* = 5; left panel), whereas in CKD mice, the period fluctuated 1–2 weeks after adenine feeding onset (*n* = 5; right panel). (Lower) The relative strength of spectral power serves as a rhythmicity measure, which showed a decreasing trend over time in CKD mice (*n* = 5; right panel) compared to controls (*n* = 5; left panel). (**C**) Time averages of the dominant period from Day 10 to Day 35 did not differ significantly between the two groups (*p* = 0.83), but the standard deviation (SD) of the dominant period over time was higher in the CKD group than in controls (** *p* = 0.0066). Consistent with unstable periodicity, the CKD group also showed lower average rhythmicity over time compared to controls (* *p* = 0.012). Horizontal dashes indicate mean values.

We created CKD model mice through continuous *ab libitum* feeding of 0.2% *w*/*w* adenine diet (see Materials and Methods). The adenine diet damaged the kidneys over the course of 5–6 weeks, and mice gradually lost robustness of rhythmicity in circadian locomotor activities under constant darkness (DD). Locomotor activity doubleplots (plotting over days *n* and *n*+1 in *n*th row and over days *n*+1 and *n*+2 in (*n*+1)th row) indicate unstable rhythmicity in CKD mice compared to controls (Figure 1A). Presented side-by-side with the doubleplots are heatmaps of a spectrogram (from day 10, due to initial effects), which visualize the detailed, parameterizable differences between the two groups (Figure 1A). In the CKD group, impairments in behavioral rhythm were manifest in three parameters: decrease of activity counts during the active phase, unstable circadian period, and disorganization of activity bouts. We quantified the latter two parameters through time-dependent spectral analysis based on sliding window Fast Fourier Transform (FFT) after smoothing, as we have done previously [23,24]. The spectrogram tracks how the dominant period of locomotor activity changes over time, and whether there is disorganization among period components. When disorganized activity bouts occur, then more activity peaks emerge over a (circa) 24-h cycle; in spectrogram, these appear as fragmented shorter-than-24 h period powers in addition to the 24-h power (Figure 1A). This disorganization can be quantified as a “rhythmicity” measure by taking a ratio of dominant spectral power over all period components, which decreases as the off-24 h period components appear (see Materials and Methods). Period and rhythmicity parameters were calculated for all samples to reveal that the loss of robustness of the circadian locomotor rhythm begins to occur in 1–2 weeks after adenine feeding (Figure 1B). Due to the unstable period in CKD animals, it was not possible to determine differences in period time-average throughout Day 10 to Day 35 (control: 23.66 ± 0.01; CKD: 23.47 ± 0.16; mean ± SEM; *n* = 5 animals for each group; *p* = 0.83, Mann–Whitney *U*-test), and the difference became significant only in the last 5 days (Day 30–35; * *p* = 0.037). However, the variability of period over time as determined by standard deviations was significantly larger in CKD animals (control: 0.31 ± 0.05; CKD: 1.06 ± 0.31; ** *p* = 0.0066) (Figure 1C, upper panel). The rhythmicity time average in CKD animals similarly reflected impaired robustness of circadian rhythm and was significantly lower than controls (control: 0.08 ± 0.001; CKD: 0.06 ± 0.005; *n* = 5; * *p* = 0.012) (Figure 1C). This difference was consistent over time and standard deviations were not statistically different (control: 0.006 ± 0.001; CKD: 0.008 ± 0.002; *p* = 0.30). Although the loss of robustness of circadian rhythmicity is clear in the adenine-fed mice, it may still not be possible to conclude that CKD impairs circadian clock mechanisms. In the following sections, we present evidence that 0.2% adenine feeding mirrors the pathophysiological signs of CKD, along with more direct signatures of circadian rhythm impairments at the tissue level.

### 2.2. Confirmation of Adenine-Induced CKD Model

**Figure 2:**
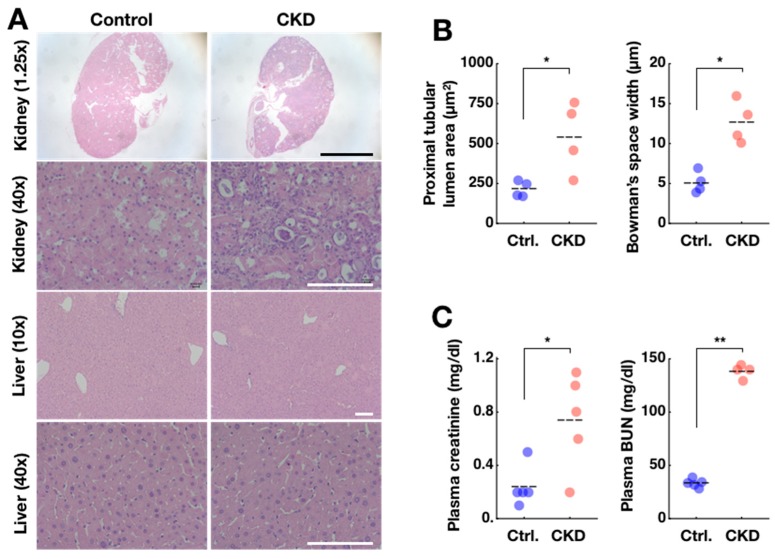
Adenine-induced CKD model is validated by histological and plasma parameters. (**A**) Structural abnormality was evident in kidneys from CKD model mice at the histological level (upper two panels) and was also evident from gross anatomical shape (see Supplementary Figure S1A,B). In livers from CKD mice, damage was not as severe (lower two panels). Objective magnification for each image is indicated in parentheses. Black scale bars in top panels indicate 1 mm. White scale bars indicate 100 µm. (**B**) Enlargements in proximal tubules and Bowman’s spaces were confirmed in CKD animals (* *p* < 0.05). Each data point indicates the average of measurements from one animal. Horizontal dashes indicate means of all animals. (**C**) Blood plasma concentrations of creatinine and BUN were also increased in CKD mice, suggesting renal failure (* *p* < 0.05; ** *p* < 0.01). The BUN level is an underestimate as the upper bound of measurement is 140 (see Materials and Methods).

After 5–6 weeks of the adenine diet, kidney damage in CKD mice was easily identifiable by the pale color seen throughout the whole organ, contrasting with the red kidneys from the control mice (Supplementary Figure S1A,B). The CKD model mice also had dirty, wet cages possibly due to proteinuria and lost weight over time compared to controls (Supplementary Figure S1). Damage to the CKD kidney was confirmed in a more detailed analysis of thin-section micrographs (Figure 2A, upper-right panels). Adenine-induced damage was most prominent in the proximal convoluted tubules, characterized by increased tubular cross-section area (control: 215 ± 25 µm^2^; CKD: 542 ± 112 µm^2^; mean ± SEM; *n* = 4 animals per group; * *p* = 0.03, Mann–Whitney U-test) (Figure 2B). In addition, distorted proximal convoluted tubule shapes could be observed in CKD kidneys, indicated by higher within-animal standard deviations of in their cross-sectional area measurements compared to controls (control: 105 ± 25; CKD: 356 ± 104; *p = 0.03). Damage was also evident in glomeruli through enlargement of the glomerular tuft areas (control: 1651 ± 59 µm^2^; CKD:2331 ± 383 µm^2^; *p* = 0.60) and a widening of Bowman’s spaces (control: 5.1 ± 0.7 µm; CKD: 12.7 ± 1.3 µm; * *p* = 0.014) in CKD kidneys (for detailed quantification procedures, see Supplementary Figure S2). Distorted renal corpuscles reflected inflammatory damage and fibrosis, consistent with histological traits in other murine CKD models [25]. The glomeruli and proximal tubules are the primary sensors and effectors in the progression of CKD. Proximal tubules are particularly vulnerable to injury because they are packed with mitochondria and depend on oxidative phosphorylation. Proximal tubules, which make up ~90% of kidney cortical mass, can lead to fibrosis, glomerulosclerosis, and CKD. The liver is sensitive to toxicity of exogenous chemicals and it also houses the strongest peripheral clock in the body. However, the damage in liver was mild (Figure 2A). Blood sample analysis from these animals supported the finding that adenine-induced damage occurred primarily in the kidney and not liver (analysis via colorimetry; see Materials and Methods). CKD mice showed elevated blood urea nitrogen (BUN) (control: 33.1 ± 1.7 mg/dl; CKD: 138.5 ± 3.2 mg/dl; ** *p* = 0.0098) and creatinine (CRE) (control: 0.24 ± 0.07 mg/dl; CKD: 0.74 ± 0.16 mg/dl; * *p* = 0.024) levels, which are signs of kidney failure (Figure 2C). Serum alanine transaminase (AST, also called GPT), a key indicator of liver failure, remained at normal levels in the same animals (control: 32.2 ± 1.8 U/I; CKD: 23.8 ± 2.7 U/I; *p* = 0.11). Together, these findings support the 0.2% chronic adenine feeding as a model of CKD. 

### 2.3. Disorganization of PER2 Expression in CKD Kidney

**Figure 3:**
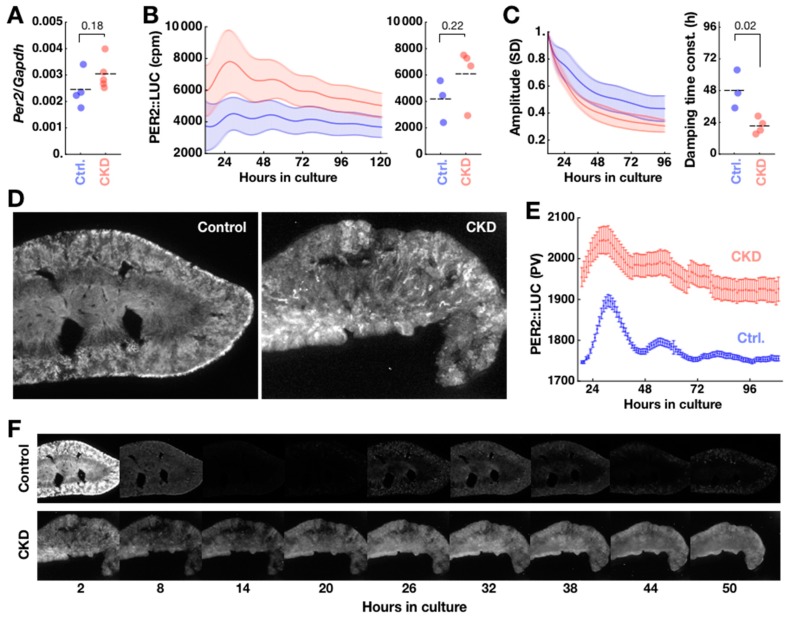
Cellular circadian clocks are disorganized in CKD kidney. (**A**) Despite histological damage, the CKD kidneys expressed *Per2* at levels comparable to control kidneys at CT20. Although the expression level appeared slightly higher in CKD kidneys, the difference was not statistically significant (*p* = 0.18). Horizontal dashes indicate averages over animals. (**B**) CKD kidneys (*n* = 4) showed a trend of higher baseline PER2::LUC expression of bioluminescence compared to controls (*n* = 3), as recorded by a photomultiplier tube (PMT) in photon counts per minute (cpm) (left). The shades of error bars indicate SEMs across samples. The time averages of these traces were not significantly different (*p* = 0.22) (right). (**C**) The amplitude of oscillations from the same traces were quantified by standard deviation (SD) in sliding 24-h window, normalized to the first timepoint of presentation (left). These revealed significantly faster damping in the CKD kidney, quantified by time constant from single exponential fitting (* *p* = 0.02) (right). (**D**) Compared to control kidneys (left panel), CKD kidneys (right panel) show tissue damage in bioluminescence images. They also acutely show PER2::LUC bioluminescence from tubular structures. (**E**) In time-lapse images, quantified by pixel values (PV), cellular circadian oscillations of PER2::LUC bioluminescence are observed in control kidneys, while high PER2::LUC background is observed in CKD kidneys. The error bars indicate standard deviation across all pixels scaled down to 10%. (**F**) Control kidneys (upper panel) show spatially organized circadian expression of PER2::LUC in culture, whereas CKD kidneys (lower panel) show disorganized and at places persistent expression of PER2::LUC.

Although renal failure was evident, this alone does not necessarily imply circadian clock failure in CKD kidneys. *Per2* is a core clock transcript that makes up the transcriptional–translational feedback loop (TTFL) of the mammalian circadian clock. In single time-point sampling qPCR of the whole unilateral kidney and liver at circadian time (CT) 20 ± 2 (mean ± SD; *n* = 5 control animals), we found no difference in *Per2* mRNA expression in *mPer2/Gapdh* transcript ratio in kidney (control: 0.0024 ± 0.0003, *n* = 4; CKD: 0.0030 ± 0.0003, *n* = 5; mean ± SEM; *p* = 0.18) (Figure 3A), as well as in liver (control: 0.0019 ± 0.0005, *n* = 5; CKD: 0.0023 ± 0.0001, *n* = 5; *p* = 0.21). The level of *Per2* expression in CKD kidney implied a necessary condition for circadian clock function. 

Detailed spatiotemporal dynamics of molecular clock activity in CKD kidneys was then tracked using explant cultures from a luciferase (LUC)-fusion protein based PER2 knock-in reporter strain (PER2::LUC; Yoo et al., 2004) sharing the same genetic background (C57/BL6J) as the wild type (WT) used in the other experiments of this study. Upon circadian expression of PER2::LUC fusion protein, light is produced through the luciferase reaction with luciferin substrate added to the culture medium. The PER2::LUC activities were generally higher in CKD kidney slices (*n* = 4 animals) than control kidney slices (*n* = 3 animals), although the difference was not statistically significant (Control 4154 ± 928 cpm; CKD 6094 ± 1077 cpm; *p* = 0.22, Mann–Whitney *U*-test) (Figure 3B). The oscillatory amplitude, as assessed by standard deviation in a sliding 24-h window, damped more than two times faster in CKD kidneys than in control kidneys (time constant of decay: control 48 ± 8 h; CKD 21 ± 3 h; * *p* = 0.02) (Figure 3C). Imaging of the kidney slice culture dramatically revealed spatially disorganized PER2::LUC expression in CKD kidneys (Figure 3D, right), which confirmed higher background PER2::LUC expression with weakened rhythmicity in CKD kidneys (Figure 3E). Normal kidney culture shows robust spatiotemporal ordering of PER2::LUC expression [17], which was reproduced in our control kidney slice culture (Figure 3D,F). In addition, we observed localized PER2::LUC activities in small blobs in the cortex of control kidneys, which had the shapes and positions of glomeruli. CKD kidney slices initially showed strong PER2::LUC activity in tubular structures which diminished after one hour in culture. While the control kidneys showed clear circadian PER2::LUC expression rhythm (Figure 3F, upper), expression rhythm was blunted in CKD kidneys with sustained PER2::LUC expression throughout the tissue (Figure 3F, lower). The overall glomerulus-like PER2::LUC activity also did not contrast in CKD kidney slices against the sustained bioluminescence background.

### 2.4. CKD Alters Kidney Clock but not SCN Clock

**Figure 4:**
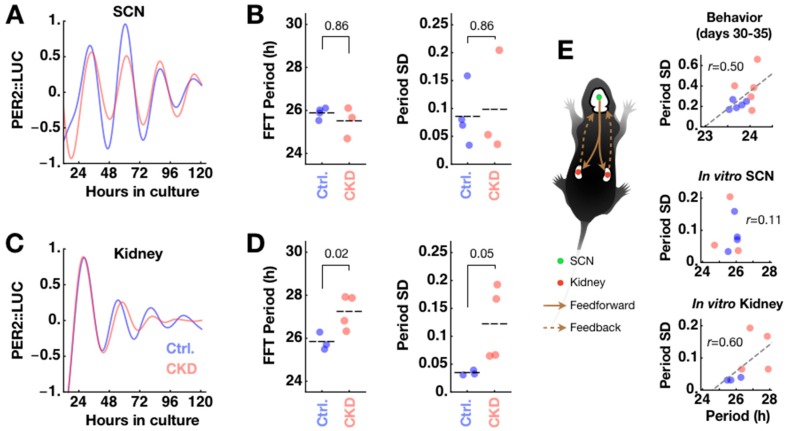
Under the CKD condition, the intrinsic suprachiasmatic nucleus (SCN) clock is unaffected while the kidney clock slows down and becomes unstable. (**A**) Isolated SCN explants from PER2::LUC mice under control (blue; *n* = 4) and CKD (red; *n* = 3) conditions maintain similar oscillations under culture. Shown are detrended and normalized ensemble averages across samples. (**B**) Their period and period standard deviation (SD) as measures of rhythm instability (see Figure 1C) are statistically indistinguishable (*p* = 0.86) between control and CKD conditions. (**C**) Kidney slices show different period and stability characteristics of oscillations under control (*n* = 3) and CKD (*n* = 4) conditions. Shown are detrended and normalized averages from Figure 3B. (**D**) CKD kidneys oscillate with a significantly longer period (* *p* = 0.02) and unstable period (*p* = 0.05). (**E**) Like other peripheral clocks, the circadian clock pacing in kidney is coordinated by feedforward control from the SCN. Under this scenario, clock feedback from CKD kidney is likely to disturb normal pacing of the SCN. Right panels are replotted for paired sets from Day 30 to Day 35 of behavioral data in Figure 1B and PER2::LUC oscillation data in Figure 4B,D. These visualize changes of period length and instability under CKD through the circadian hierarchy. The gray dashed line indicates linear regression over all samples; *r* indicates Pearson’s correlation coefficient.

The period and phase of the behavioral circadian rhythm are determined by the interaction of the SCN clock with feedback signals from peripheral clocks. A peripheral clock may be damaged under disease conditions, meaning that its feedback can disturb the master clock’s pacemaking, resulting in abnormal circadian behaviors. We previously found that co-culture with CP can speed up the circadian clock of the SCN close to the behavioral circadian period under constant darkness [8]. The feedback interaction from the kidney clock to the SCN clock is difficult to assess in the same in vitro setting because, in our experimental condition, the periods were similar in the SCN (25.88 ± 0.12 h; *n* = 4) and the kidney (25.82 ± 0.24 h; *n* = 3; *p* = 0.81; Student’s *t*-test). However, we found that CKD caused the kidney clocks to deviate from normal timekeeping but left the SCN clocks unharmed. SCN explants from CKD animals maintained the same rhythms compared to SCN explants from control animals in isolated culture (Figure 4A) in terms of period (CKD 25.49 ± 0.41 h; *n* = 3; *p* = 0.86) and period standard deviation as an instability measure (control 0.086 ± 0.026; CKD 0.098 ± 0.054; *p* = 0.86) (Figure 4B). In contrast, kidney explants from CKD animals expressed altered PER2::LUC rhythms (Figure 4C). CKD kidneys maintained a significantly longer period rhythm (27.23 ± 0.39 h; *n* = 4; * *p* = 0.02) and unstable period over time (control 0.034 ± 0.002; CKD 0.123 ± 0.033; *p* = 0.05) (Figure 4D). The SCN is undoubtedly the origin of circadian rearrangement of the body’s internal clocks though feedforward signaling, but it is also subject to modulation by multiple feedback pathways (Figure 4E). It is interesting to note that the kidney circadian clock slows down under CKD, which may be comparable to the slight period increase in the later phase of adenine feeding (Day 30–35; control 23.72 ± 0.16 h; CKD 23.99 ± 0.20 h; * *p* = 0.037; Figure 1B). These results raise the possibility that the kidney clock forms an important feedback pathway to the master clock, namely the SCN. 

It should be noted that the SCN is already a robust clock and additional feedback interaction from the periphery does not imply that the peripheral clocks are additional clock components of the SCN. These interactions are not analogous to the case of synchronization in weakly coupled oscillators, and it is difficult to expect complete synchronization of rhythms across all circadian clocks in the body. What is likely to happen is the spontaneous coordination of relative phases and periods of bodily clocks, similar to phase reorganization among cellular circadian clocks in the SCN [26,27]. However, it is interesting that the SCN and kidney clocks have similar periods under normal conditions. The similar periods are required for circadian feedback to strengthen robustness of the master clock. Within the brain environment, feedback from the CP clock is more straightforward to interpret: the SCN clock speeds up when co-cultured with the CP to a level comparable to the behavioral circadian period. Since the CP can influence the SCN clock via diffusion through cerebrospinal fluid (CSF), the finding easily translates to in vivo and targeted *Bmal1* silencing in the CP, resulting in a longer circadian free-running period [8]. A comparable experimental design is difficult for the kidney-SCN clock interaction, as the blood-brain barrier isolates the brain from blood plasma. Moreover, complete isolation of the kidney clock is challenging since the kidney is composed of heterogeneous cell types with diverse transcriptomes, limiting the Cre recombinase approach.

## 3. Discussion

CKD, which is increasing in global prevalence and health-care burdens, is characterized by a progressive decline in the glomerular filtration rate (GFR). Sleep disturbances are much more prevalent in CKD and dialysis patients than in the general population [28], and comorbidities are common in patients with CKD. Previous studies found decreased sleep efficiency, a higher arousal index, and increased sleep apnea in dialysis patients [29,30]. Although the etiology is unclear, there are indications that an impaired circadian clock is involved in sleep disturbances. In human patients, circadian blood pressure rhythms were disturbed under unilateral nephrectomy [31]. In rats, altered circadian gene expression profiles were found in the hypothalamus containing the SCN after 5/6 nephrectomy (2/3 nephrectomy one kidney and whole nephrectomy on the other kidney) [20]. Circadian rhythms manifest through physiological rhythms such as heart rate variability (HRV) and blood pressure (BP). Consistent evidence exists for renal transplantation normalizing HRV [32] and rescuing non-dipping BP [19], indicating that the kidney plays a role in the normal maintenance of circadian rhythms in the body.

The behavioral circadian period and period stability are well-defined parameters in the inbred C57BL/6 strain that enabled forward genetics approaches to mammalian circadian biology. A relationship between mood conditions and circadian rhythms has been sought in the same strain, demonstrating that the mouse strain is capable of connecting behavioral circadian rhythms with internal states at molecular and cellular levels. However, it is difficult to operationally define a disease condition in a similar approach. The advantage of CKD model is that the disease condition is relatively more specific and localizable to the kidneys; concurrently, we find circadian rhythms disruptions in the CKD but not in the SCN.

There are two proposed mechanisms for adenine-induced CKD: DHA crystal deposition; and impairment in arginine vasopressin (AVP) V2 receptor signaling. Under physiological conditions, adenine is absorbed by the intestine and metabolized by adenine phosphoribosyl transferase (APRT) to AMP and adenine derivatives. One of the adenine derivatives, xanthine, is ultimately excreted by the kidney after being converted to uric acid. However, with pharmacological doses, adenine can be metabolized by Xanthine oxidase to 2,8-dihydroxyadenine (DHA), which can form crystals that are deposited in renal tubulointerstitium and cause renal tubular injury [22]. As a signaling molecule, adenine can act directly on the renal tubule by interfering with AVP V2 receptors and the cAMP/PKA-dependent pathway, which causes downregulation of aquaporin 2 (AQP2) in the collecting duct and NKCC2 in the medullary thick ascending limb of the loop of Henle. As a result, adenine fed mice show early pre-renal failure that can lead to chronic kidney disease [33].

Kidney also has its own circadian clock that determines timing of ion channel/transporter expression and hormone release. It has a molecular feedback network of clock gene expression for autonomous timekeeping [34]. In isolation, kidney explant cultures maintain endogenous circadian rhythms in expression of *Per1,2*, the core clock transcripts [5,6]. The circadian clock directly regulates expression of proteins for water homeostasis. Expression of sodium proton exchanger (NHE3) is circadian-controlled through E-box [35]. Renal epithelial sodium channel (αENaC) expression, critical for sodium balance, is decreased upon silencing of *Per1* expression [36]. Nephron-specific *Bmal1* knockout mice exhibit increased urine volume in what appears to be a shift in the circadian rhythm of urinary sodium excretion [15]. Moreover, the expression level of a prominent water channel in conducting ducts, AQP2, correlates with a day/night cycle in diuresis, with urine levels higher during the day and lower at night [37]. These suggest that circadian rhythmic water homeostasis is a self-controlled process within kidney, and is consonant with a sustained circadian osmosis rhythm in cultured kidney explants [17]. 

The general imbalance of water and ions resulting from kidney failure can have unspecified effects on sleep. During dialysis treatment, disequilibrium syndrome can cause osmolality changes and paradoxical acidosis in CSF, leading to cerebral edema and sleep disturbances [38]. In the last stage of CKD, also termed ESRD, sleep disturbances can be attributed to an organic brain syndrome. Gradual reduction in renal function can lead to uremic encephalopathy through chronic hypertension and electrolyte imbalance. Hypertension often leads to a reversible posterior leukoencephalopathy syndrome, with vasogenic edema developing in and around the brainstem. Underlying sleep rhythm is the circadian rhythm, and it is also likely that disrupted rhythmic homeostasis affects rhythmic expression of circadian clock genes. One potential pathway is salt-inducible kinases 1 [39] and 3 [40], which induce expression of *Per* and can be an entry route for external information about the body’s metabolic state to the circadian clock.

The current study is proof of concept for the role of reverse hierarchical feedback from peripheral clocks. There can be many routes, including metabolic pathways, that provide the feedback to the central clock [41]. Our adenine-induced CKD model provides evidence that, unlike what is expected from a simple hierarchical organization of circadian clocks, overall circadian rhythm manifested by locomotor activities can weaken when a major circadian clock in the periphery is compromised. It was originally found that the phase ordering of peripheral circadian clocks has organized structure and that this becomes disorganized when the SCN is ablated [42]. Yet, the master clock’s orchestration of such circadian structure can be replaced by liver in the absence of the SCN, through regularly timed feeding cues [43]. The damaging effect of kidney failure on circadian clock coordination deserves clinical attention, as it can trigger a vicious cycle of damage. Circadian rhythm disruption can, in return, exacerbate renal disease condition [44]. 

For developing a treatment strategy, it will be necessary to identify the exact mechanism of circadian feedback signaling. In the future, it will be important to clarify whether the circadian pathology in CKD is attributable to the absence of rhythmic signals from the kidney or to baseline changes of plasma and CSF compositions caused by kidney failure. Such findings could open up new avenues for treating sleep disturbances common to late stage CKD patients and preventing further aggravation of kidney damage. For now, our evidence of probable retrograde clock feedback from the kidney lets us rethink the hierarchy of circadian clocks in the body, and the role of peripheral clocks in shaping sleep timing and maintenance.

## 4. Materials and Methods

### 4.1. Animals

Adult (10–20 weeks old) male C57BL/6J (National Laboratory Animal Center, NLAC, Taipei, Taiwan) and PER2::LUC [6] under C57BL/6J background (Jackson Laboratory, Bar Harbor, ME) were maintained in a temperature and humidity controlled animal room (breeding: 21.8 ± 0.4 °C, 59 ± 3%; experiment: 20.5 ± 0.4 °C, 57 ± 5%; mean ± SD) under a 12:12 light:dark (L:D) cycle (light on at 7:00 h; off at 19:00 h) before being transferred to constant darkness (DD) for monitoring of circadian freerunning rhythm. All mice were habituated to a powdered normal diet (LabDiet, St. Louis, MO, USA) for one week prior to adenine diet feeding. A 0.2 % (*w*/*w*) adenine diet was prepared by adding 2 g of adenine (Sigma-Aldrich, St. Louis, MO, USA) to 1,000 g of the powdered diet. To create the adenine-induced CKD model, mice were given the adenine diet for 5–6 weeks [22]. Control mice were continuously fed with powdered normal diet. All animal protocols used in this study were approved by the Institutional Animal Care and Use Committee (IACUC) of Taipei Medical University Laboratory Animal Center. IACUC protocol numbers were: LAC-2017-0264 (approval date: November 14, 2017), LAC-2017-0469 (9 February 2018), and LAC-2018-0369 (3 January 2019).

### 4.2. Locomotor Activity Measurement

Animals were housed individually in custom-made, light-sealed boxes with computer-controlled LED lighting and a ventilator fan, as previously described [23]. Food was given *ab libitum*. Inside the box, the light intensity (156 ± 13 lux; mean ± SD) was set to match the level in the breeding rooms (153 ± 67 lux) using a current divider circuit. Outside the box, the animal room was lit only with safety lights (14 ± 2 lux) and computer monitors were covered with safety film (light level on the work table with monitors on: 15 ± 2 lux). Circadian locomotor activity from each mouse was continuously monitored by a passive infrared (PIR) motion sensor with a time resolution of 1 min. Scheduled light control and data acquisition were performed using an Arduino Mega 2560 microcontroller and a computer using custom-made software. Animals were habituated in the box under 12:12 L:D cycles (on at 7:00 h; off at 19:00 h) for a minimum of one week before entering constant darkness. The adenine diet was administered under constant darkness and locomotor activities were monitored throughout under the same conditions. Mouse weight was measured under the dim safety lights. Exact circadian time (CT) of tissue sampling was determined post hoc from locomotor activity double plots in control mice.

### 4.3. Spectral Analysis of Locomotor Activities and Circadian Heatmap

Time-dependent period and rhythmicity were calculated using a sliding window Fast Fourier Transform (FFT) to create a circadian heatmap, as done previously [23,24]. One-min resolution locomotor activity data were first smoothed using a Hodrick–Prescott (HP) filter (penalty parameter *λ* = 5.184 × 10^7^) and detrended with a heavily HP filtered trend of the activity (*λ* = 5.184 × 10^9^). From the smooth detrended data, spectral components were decomposed with FFT in a 7-day sliding window. The first 10 days of recording were excluded due to initial jittering and boundary effects of smoothing and detrending. The final converted data were presented as a spectrogram: a power spectrum over a period range (reciprocal of frequency) for each time point. The spectrogram was represented in a heatmap using Mathematica’s Rainbow color function. We used the spectral method because there were multiple embedded fundamental periods outside the 24-h period scale. The ratio of peak power (*S_p_*) of the circadian period (*T*) over the sum of all power (*S_i_*) was used as a quantitative measure of rhythmicity as the following equation. The spectral power used to calculate rhythmicity was not normalized.

(1) $$\mathrm{Rhythmicity}\;=\;\frac{S_{p}}{\sum_{i}^{N}S_{i}}$$

The dominant period was the period of the peak power, which was found for each time point and its standard deviation over time was used as a secondary, inverse measure of rhythmicity.

### 4.4. Thin-Section Micrographs and Histological Analysis

Kidneys and liver samples from each of the four major lobes were taken from control and CKD mice immediately after completion of locomotor activity measurements, approximately at CT20, and stored at −76 °C. Samples were sent to Bio-Check Laboratories Ltd. (New Taipei City, Taiwan) for preparation of thin section (3 μm thickness) and hematoxylin and eosin (H&E) staining. The thin sections were scanned on an Olympus IX81 microscope (Olympus, Tokyo, Japan) at objective magnifications of 10× and 40×. Histological image analysis was performed manually by two researchers using ImageJ (National Institutes of Health, Bethesda, MD, USA). Measurements included Bowman’s space width, glomerular turf area, and tubular proximal lumen area. All images were firstly converted into 32-bit grayscale and all pixels were calibrated to corresponding µm length using a pre-calibrated scale embedded on the image (Figure S2B). In three 10× images of kidney thin sections, we identified three clear glomeruli and measured the largest width of empty space and determined it as Bowman’s space width. For each glomerulus, the freehand selection tool was used to measure the tuft area. Nine tubular proximal lumen areas from three different 40× images were measured (Supplementary Figure S2). Identification of these structures was based on Junqueira’s Basic Histology Atlas [45]. The mean value of the respective measurements was calculated for each mouse.

### 4.5. Sampling and Analysis of Blood Serum

We collected blood (~500 μL) by facial vein puncture at the time of tissue sampling at around CT20. Serum was separated by centrifugation at 3000 rpm for 5 min and was sent to Taiwan Animal Consortium (Taipei, Taiwan) for biochemical analyses. Analyses were performed using Fuji Dri-Chem Clinical Chemistry Analyzer FDC 3500 (FujiFilm, Tokyo, Japan), which uses colorimetry and electrolyte measurement (https://www.fujifilm.eu/fileadmin/migration_uploads/FUJI_DRI-CHEM4000i.pdf). Blood urea nitrogen (BUN) level that exceeded measurement precision was indicated “>140 mg/dl” in the analysis report and this upper cap was used as-is in statistical analysis since the value is much beyond the normal range. Compared to enzymatic or high-performance liquid chromatography (HPLC) methods, the colorimetric method used in this study can overestimate the absolute concentration of creatinine (CRE) [46]. We have therefore used estimated CRE as a relative measure only.

### 4.6. Total RNA Isolation

Unilateral kidneys and liver tissues not used for histology were quickly sampled on ice and preserved in RNAlater (Ambion/Thermo Fisher Scientific, Waltham, MA, USA) prior to snap freezing in liquid nitrogen. SCN and CP samples were dissected from the brain in ice-cold Ca^2+^ and Mg^2+^-free HBSS (Gibco/Thermo Fisher Scientific, Waltham, MA, USA) as previously described [8]. Whole kidneys were used after removal of adrenal glands. Liver samples were taken from all four major lobes of each liver. Sampled tissues were homogenized and total RNA was purified using TRIzol (Ambion; 500 μL/sample). 100 μL DEPC water was used for elution of each sample and stored at -76 °C until further analysis. Purity and concentrations of samples were checked using a NanoDrop 2000 spectrophotometer (Thermo Fisher Scientific, Waltham, MA, USA).

### 4.7. Quantitative Real-Time Polymerase Chain Reaction (RT-qPCR)

After determination of RNA concentration using a NanoDrop 2000, total RNA was adjusted to 2 μg in kidney and liver samples in 10 µL DEPC water, respectively. cDNA was synthesized using random primers and High-Capacity cDNA Reverse Transcription Kits with RNase Inhibitor (Thermo Fisher Scientific) in 20-µL reactions. For RT-qPCR, cDNA was diluted to 50 ng in PowerUp SYBR Master Mix (Thermo Fisher Scientific) in a total volume of 10 µL and quantified in technical triplicates on QuantStudio 3 real-time PCR system (Thermo Fisher Scientific). Sequences of the forward and reverse primers used were as previously used (Myung et al., 2015) (purchased from Integrated DNA Technologies): *Gapdh* forward ACGGGAAGCTCACTGGCATGGCCTT, *Gapdh* reverse CATGAGGTCCACCACCCTGTTGCTG (amplicon length 311bp); *mPer2* forward GGCTTCACCA TGCCTGTTGT, *mPer2* reverse GGAGTTATTTCGGAGGCAAGTGT (206bp).

### 4.8. Explant Culture

Animals were anesthetized with isoflurane and sacrificed under dim light (43 ± 10 lux; mean ± SD) during their subjective daytime. Brains and kidneys were quickly removed and immersed in ice-cold HBSS (Gibco). Paired sets of the SCN and kidneys were isolated from the same animals. For imaging and luminometry, kidneys were sliced at 50–100 μm thickness on a vibratome (Leica VT1000S, Heidelberg, Germany). For luminometry, the whole SCN was carved out in ice-cold HBSS under a surgical microscope (Nikon SMZ745T, Tokyo, Japan) using cut blades as described previously [8]. Each explant was transferred to culture membrane insert (Millicell-CM, Millipore, Bedford, MA, USA) and cultured at 37 °C in a transparent 35-mm dish (Corning, Corning, NY, USA) or 35-mm glass-bottom dish (Alpha Plus Scientific, Taoyuan, Taiwan), with the lid sealed with High Vacuum Grease (Dow Corning, Midland, MI, USA). Explants were cultured in B27-supplemented Neurobasal-A (Gibco) medium containing: 4.2 mM sodium bicarbonate (Gibco), 10 mM HEPES (Gibco), 1% GlutaMAX (Gibco), 1% penicillin-streptomycin (10 U/μL penicillin and 10 μg/μL streptomycin, Gibco), 2% B-27 (Gibco), and 300 μM beetle luciferin (VivoGlo P1043, Promega, Madison, WI, USA), at pH 7.3 at 37 °C before addition of B-27 and antibiotics.

### 4.9. Bioluminescence Imaging and Luminometry

PER2::LUC is a luciferase fusion protein that reports its expression bioluminescently. Time-dependent changes in PER2::LUC expression were measured in real-time via a cooled-CCD camera (imaging) or a photomultiplier (luminometry). Bioluminescence imaging was performed in a previously described custom-made, light-sealed inverted microscope system [26], which has a simplified optical path optimized for low-light imaging, with a 4× objective lens and a 0.35× relay lens (Olympus). The cultured sample was maintained at 37 °C in a stage-top incubator (Tokai HIT, Shizuoka, Japan), and time-lapse imaged with an Orca R2 cooled-CCD camera (Hamamatsu Photonics, Hamamatsu, Japan) with external water-cooling (20 °C; TGV-10, Taipei, Taiwan). Images were taken under 1 h exposure at 4×4 binning, at a sampling interval of 1 h. For luminometry, an equal number of control and test samples were measured on the same 8-dish wheel in Kronos Dio (ATTO, Tokyo, Japan); light from each dish was measured under 1 min exposure at sampling interval of 10 min, with the nominal temperature of 37 °C and actual temperature maintained between 37.6–37.7 °C, measured with iButton datalogger in the dish (DS1921L, Maxim Integrated, San Jose, CA, USA). Bioluminescence traces were detrended using the HP filter as described previously [23].

### 4.10. Statistical Analyses

All data analyses including statistics were performed in Mathematica (Wolfram Research, Campaign, IL, USA). Statistical significance was determined for pairwise differences using the Mann–Whitney *U*-test, unless specified otherwise. Student’s *t* test was used in parallel and stated as such when normality was generally known for the measured parameter. All *p* values were stated as-is; in graphs, *p* < 0.05 is indicated with a single asterisk (*) and *p* < 0.01 is indicated with double asterisks (**) as a guide. All data points were indicated in graphs. When pairwise differences were tested, we indicated the standard error of the mean (SEM) and when intrinsic variability was considered, we stated the standard deviation (SD) in the text.

## Supplementary Materials

Supplementary materials can be found at https://www.mdpi.com/1422-0067/20/11/2765/s1.

## Abbreviations

SCN	suprachiasmatic nucleus
CP	choroid plexus
ESRD	end-stage renal disease
CKD	chronic kidney disease
FFT	Fast Fourier Transform
PER2	PERIOD2
WT	wild type
CSF	cerebrospinal fluid
CT	circadian time
