# Supplementary material for: The Kidney Clock Contributes to Timekeeping by the Master Circadian Clock
Source: Int J Mol Sci. 2019 Jun 5;20(11):2765. doi: 10.3390/ijms20112765 (PMC6600447; doi:10.3390/ijms20112765)

Supplementary Figures

Figure S1. Gross anatomical photos of kidneys from control and CKD mice.
In contrast to kidneys from control mice (**A**), kidneys from CKD mice are visually distinct and identifiable by their pale color (**B**). Shown are representative examples (indexed #1-6). Compared to the cage housing a control mouse (**C**), the cage that houses a CKD mouse remained wet possibly due to proteinuria (**D**). (**E**) The CKD model mouse also lost weight gradually after adenine was added to the diet. Traces show group averages and error bars indicate standard deviations. *n*=5 per group.

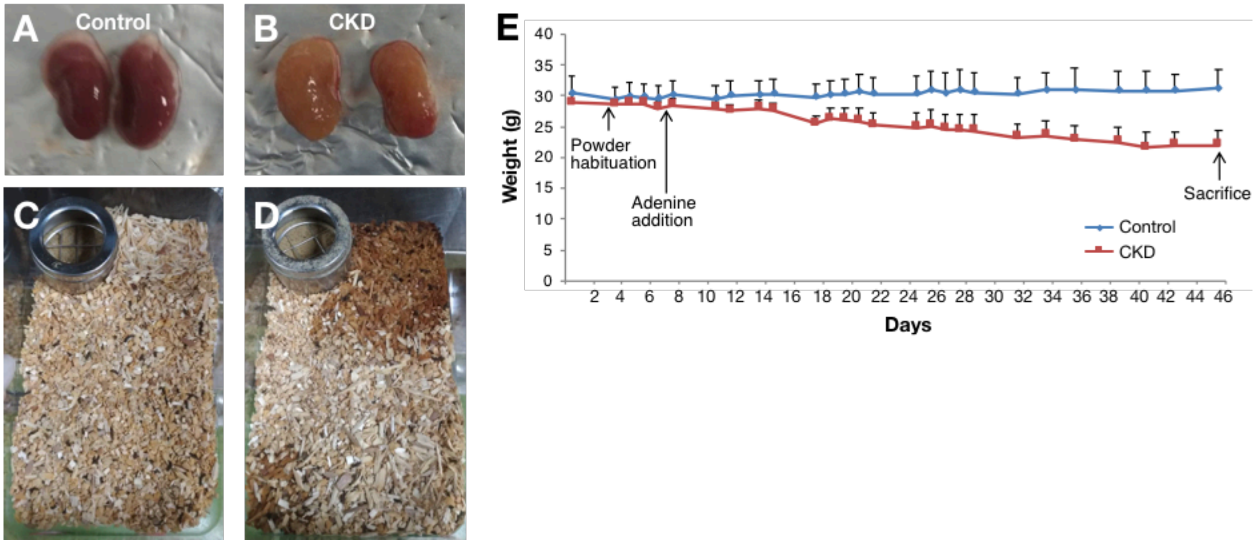

Figure S2. Example of the histological image analysis process.
These panels explain the process of histological quantification from a CKD kidney example. After the image was converted to grayscale (**A**), the size was calibrated to the pre-calibrated scale bar stamped on the image (**B**), affected glomeruli were identified (**C**), and length and area were measured (**E-F**) using ImageJ. Shown is a kidney section from a CKD mouse (#1-10). For details, see the subsection "Thin-section micrograph of kidney and histological analysis" in Materials and Methods. Scale bars indicate 100μm for 10x images and 10μm for 40x, respectively.

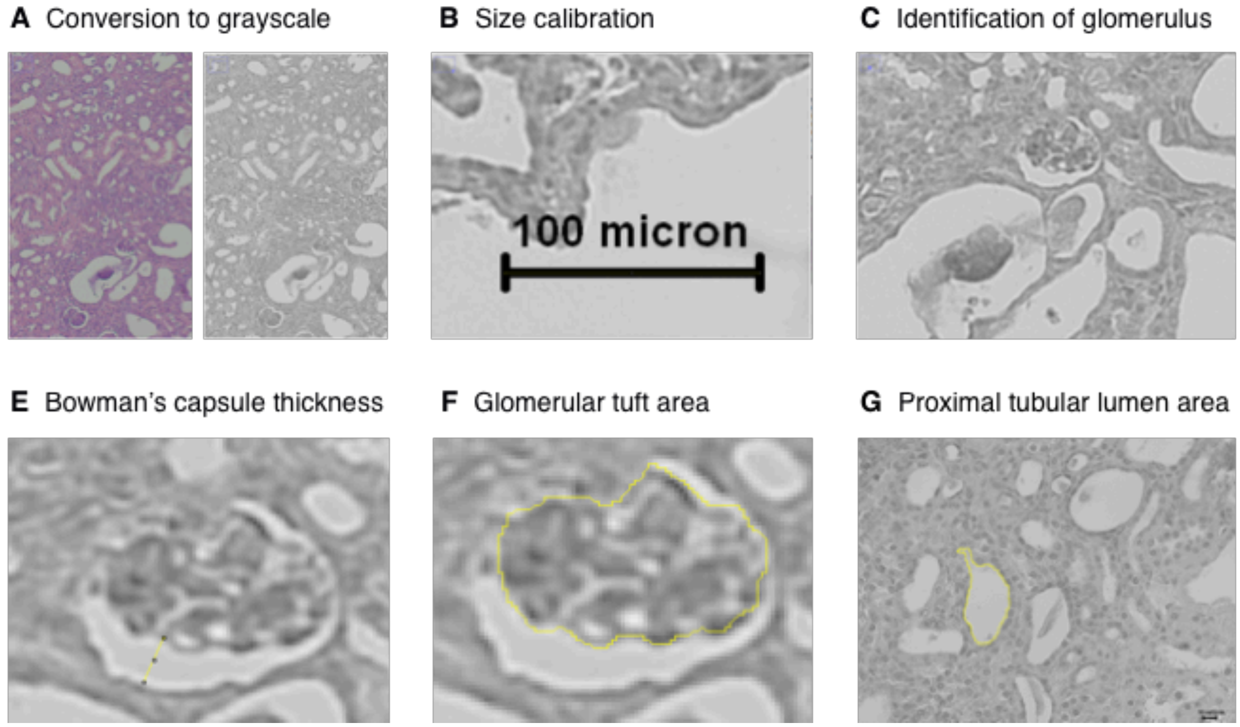

Supplement: Supplementary file 1 [file ijms-20-02765-s001.pdf]
